# Optimizing Inter-Professional Communications in Surgery: Protocol for a Mixed-Methods Exploratory Study

**DOI:** 10.2196/resprot.3623

**Published:** 2015-03-05

**Authors:** Julie Hallet, David Wallace, Abraham El-Sedfy, Trevor NT Hall, Najma Ahmed, Jennifer Bridge, Ru Taggar, Andy J Smith, Avery B Nathens, Natalie G Coburn, Lesley Gotlib-Conn

**Affiliations:** ^1^Sunnybrook Health Sciences CentreDivision of General SurgeryToronto, ONCanada; ^2^University of TorontoDepartment of SurgeryToronto, ONCanada; ^3^Sunnybrook Research InstituteToronto, ONCanada; ^4^Sunnybrook Health Sciences CentreQuality and Patient SafetyToronto, ONCanada; ^5^Saint-Michael’s HospitalDivision of General SurgeryToronto, ONCanada

**Keywords:** communication, interprofessional, pager, resident, nurse, education, patient safety

## Abstract

**Background:**

Effective nurse-physician communication is critical to delivering high quality patient care. Interprofessional communication between surgical nurses and surgeons, often through the use of pagers, is currently characterized by information gaps and interprofessional tensions, both sources of workflow interruption, potential medical error, impaired educational experience, and job satisfaction.

**Objective:**

This study aims to define current patterns of, and understand enablers and barriers to interprofessional communication in general surgery, in order to optimize the use of communication technologies, teamwork, provider satisfaction, and quality and safety of patient care.

**Methods:**

We will use a mixed-methods multiphasic approach. In phase 1, a quantitative and content analysis of alpha-numeric pages (ANP) received by general surgery residents will be conducted to develop a paging taxonomy. Frequency, timing (on-call vs regular duty hours), and interval between pages will be described using a 4-week sample of pages. Results will be compared between pages sent to junior and senior residents. Finally, using an inductive analysis, two independent assessors will classify ANP thematically. In Phase 2, a qualitative constructivist approach will explore stakeholders’ experiences with interprofessional communication, including paging, through interviews and shadowing of 40 residents and 40 nurses at two institutions. Finally, a survey will be developed, tested, and administered to all general surgery nurses and residents at the same two institutions, to evaluate their attitudes about the effectiveness and quality of interprofessional communication, and assess their satisfaction.

**Results:**

Describing the profile of current pages is the first step towards identifying areas and root causes of IPC inefficiency. This study will identify key contextual barriers to surgical nurse-house staff communication, and existing interprofessional knowledge and practice gaps.

**Conclusions:**

Our findings will inform the design of a guideline and tailored intervention to improve IPC in order to ensure high quality patient care, optimal educational experience, and provider satisfaction.

## Introduction

### Background

#### Interprofessional Communication Challenges

Effective interprofessional communication (IPC) is associated with greater patient safety, quality of care, and provider satisfaction. Ineffective nurse-physician communication has been linked to medication errors, patient injuries, and patient deaths [1]. Despite advances in clinical communication technologies designed to facilitate communication, there is limited evidence suggesting that any have led to improvements in the ability of health professionals to communicate effectively [2]. Communication systems designed for interprofessional use are not always optimal from the perspectives of the different providers who use them. For instance, the introduction of alphanumeric paging in surgery has been reported to pose a number of problems with communication from surgeons’ perspectives, including insufficient content in a page and limited indication of the degree of urgency [3]. In addition, communication between nurses and physicians is inherently challenged by profession-specific communication styles and norms which create barriers to interprofessional care [4]. An abundance of research indicates a range of barriers to nurse-physician communication, which are embedded in professional cultures and power dynamics [5].

In contrast to other areas of inpatient care, direct face-to-face communication between nursing and surgical staff is challenged by the need for house staff presence in the operating room, in the emergency department, and attending to off-service patients. At this time, paging remains the primary mode of electronic communication between surgical nurses and house staff. Because of a lack of consensus guidelines for sending and responding to a page, nurses’ decision to page surgeons for patient care, and surgeons’ responses rely on each professional’s judgment. Tension and communication breakdown between nurses and surgeons can arise when either party perceives a problem with a paged communication.

#### Impact of Inefficient Communication

Both nurses and surgeons have indicated that the current IPC model based on paging is a source of workflow interruption, particularly when sending or receiving pages seems incessant by either party [6,7]. Such paging systems are indeed disruptive for all health professionals [8]; during peak periods of the day, pages are received by general surgery house staff as often as every 6 to 12 minutes [9]. Of these frequent pages, up to 50% result in patient care interruption, including 19% interrupting direct patient contact [6,10,11]. Previous assessments of paging patterns revealed that 34% of pages received by residents were judged to require a response within one hour and result in change in patient care. Improving communication patterns and reducing unnecessary pages could result in 42% fewer interruptions in patient care [12].

Such workflow interruptions have been reported as detrimental for patient care. A direct observation study of ward nurses indicated that each interruption during medication administration and procedures is associated with a 13% increase in clinical errors [13]. Disruption of clinical work has also been related to more frequent surgical errors, with teamwork and communication failures identified as main predictors of these errors [14].

Negative impacts of inadequate paging systems have also been noted for educational experience of residents [15]. Up to 35% of residents’ time is filled with activities without perceived educational value, including responding to unnecessary pages, and residents have expressed the wish to improve the efficiency of paging communications to reduce work interruptions that contribute to medical errors, stress, and fatigue [15-17]. Impaired nurses’ job satisfaction due to inefficient paging communication has also been observed [18].

#### Need for in-Depth Assessment

Suboptimal use of the paging system, either through inappropriate activation/nonactivation of a page or inappropriate response to a page, leads to work interruption, communication gaps, and provider frustrations. Efforts to improve the quantity and quality of paging communications are ongoing but are challenged by poor understanding of barriers to effective IPC. A comprehensive understanding of the nature of interprofessional collaboration and communication in general surgery, including use of the existing paging system and other modes of communication, is needed to inform the development of meaningful and effective solutions to optimize interprofessional care delivered by nurses and surgeons.

### Study Purpose

We plan to describe the current paging communication system in general surgery, and to explore barriers and enablers to effective use of paging in order to optimize interprofessional collaboration, provider satisfaction, and quality and safety of patient care. This study will be carried out with the following objectives: (1) to develop a paging taxonomy, by describing the urgency and content of pages sent on a general surgery service; and (2) to explore current patterns of communication between general surgery nurses and house staff, and compare them between two institutions.

## Methods

### Study Setting

**Figure 1:**
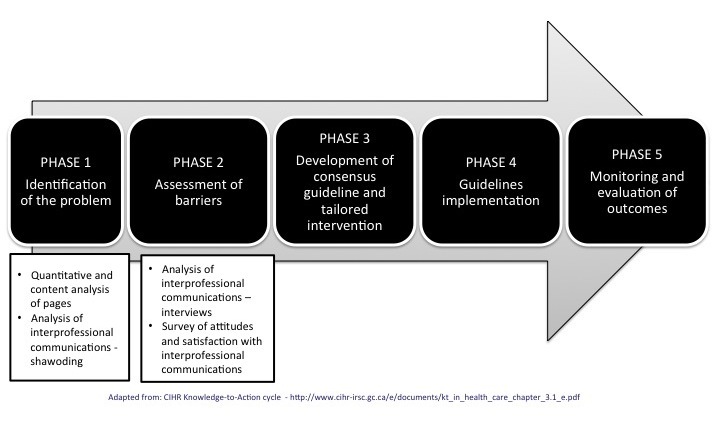
Outline of an initiative to improve interprofessional communication in general surgery.

This study is the first part of an ongoing initiative to develop, implement, and evaluate a guideline and tailored strategy to enhance IPC, in order to optimize the delivery of timely high quality patient care. The outline of this initiative is based on the Knowledge-to-Action framework, with the current study aiming at identifying the problem at hand and addressing barriers to implement solutions, before future efforts are dedicated to development and implementation of a larger, tailored intervention, and evaluation of its outcomes (Figure 1). The Sunnybrook Health Science Centre (SHSC) Research Ethics Board has approved this study (REB 044-2014).

This study will be carried out at SHSC and Saint-Michael’s Hospital (SMH) in Toronto, Ontario. SHSC is a large academic institution including a tertiary level 1 Trauma Centre and the sixth largest cancer center in North America. SMH is a large tertiary institution that also includes a level 1 trauma center. general surgery house staff covers surgical oncology, vascular surgery, acute care surgery, and trauma surgery services. In-house on-call teams include a junior resident, a senior resident, and a trauma team leader. All general surgery house staff are provided by the institution with an alphanumeric pager device associated with a unique messaging ID. Alphanumeric pages (ANP) are sent using an online tool on the hospital’s intranet through which health care professionals can type and deliver their own messages.

### Quantitative and Content Analysis of Pages

We will conduct a retrospective quantitative content analysis of pages received by residents on general surgery services at SHSC.

All pages received by residents on the general surgery service at SHSC during four consecutive weeks will be included in the study. Residents on service during this period will be identified through the hospital’s administrative records. Pages will be abstracted electronically from the pages database maintained by the Department of Information and Telecommunication, using the unique messaging ID of each resident. Based on previous exploratory analyses, we estimate that approximately 3000 pages will be retrieved.

The information retrieved will include date, time, receiver characteristics, origin (call-back number), and complete alphanumeric message for each page. Level of training of receivers will be inputted using the general surgery administrative list for the time period considered. The data will be anonymized and inputted into a spreadsheet for analysis.

The primary outcome will be the communication priority of pages based on required page-to-intervention time, divided into immediate (0-5 minutes), high (5-30 minutes), medium (30-120 minutes), and low priority (more than 120 minutes), as adapted from the institutional Escalation of Care Policy (Multimedia Appendix 1). Two independent physician assessors will code pages based on perceived priority. Disagreements will be solved by consensus or by a third physician if consensus cannot be reached.

Secondary outcomes will include number, timing, and interval of pages. Number of pages will be computed as number of pages per resident per day and number of pagers per day for the whole group of residents. Timing will consist of subdivision of pages into the following time periods: on-call (week days 5:00 pm to 7:00 am, and weekends/holidays 7:00 am to 7:00 pm), and regular duty (week days 7:00 am to 5:00 pm). Interval will be defined as time (minutes) between two pages.

We will finally proceed with content analysis of the ANP using an inductive process by two independent assessors.

We will conduct a descriptive analysis using mean with standard deviation or median with interquartile range for continuous variable, proportions with 95% confidence intervals for categorical variables. Communication priority, number, timing, interval, and content of pages will be compared between pages sent to junior (postgraduate years 1 to 2) versus senior (postgraduate years 3 to 7) trainees, using chi-square test for categorical data and students *t* test for continuous data. Inter-rater agreement between physician and nurse assessors for communication priority will be computed using a weighted kappa score [19]. All *P* values less than .05 will be considered statistically significant.

We will perform a multivariate analysis to identify factors associated with immediate/high communication priority of pages using logistic regression modeling, including variables of page timing, origin, receiver, and content category. Using the Wald chi-square test, any variable with a *P* value less than .05 will be considered a significant predictor of emergent/urgent pages.

### Exploration of Interprofessional Collaboration and Communication: a Comparative Case Study

In phase 2, we will explore general surgery nurses and house staff perspectives and experiences with regard to activating, receiving and responding to pages, and compare both groups’ opinions on when, why, and how paging should be used for communication. We will also compare the perspectives and experiences of nurses and house staff across two divisions of general surgery at the university (SHSC and SMH).

We will use a qualitative constructivist approach [20] to understand providers’ perspectives and experiences with interprofessional communication. This approach, rooted in the interpretivist paradigm [21], assumes that multiple viewpoints are operating in the construction of providers’ experiences and understandings of interprofessional communication and collaborative care. This approach aims to disentangle and explore these experiences and viewpoints accepting each as valid and true.

A theoretical sampling approach will be used. Nursing team leaders and nurses providing direct patient care will be recruited. We will recruit nurses of varying years of experience to capture the potential range of perspectives among more seasoned and junior nurses, and residents in different years of training (juniors/seniors). A total of 10 nurses and 10 residents will be recruited for interviews at each site, and an additional 10 nurses and 10 residents will be recruited for shadowing at each site.

Semistructured interviews and shadowing techniques will be used to understand nurses’ and residents’ perspectives and experiences with paging in general surgery. For nurses, the interviews will explore their perspectives on interprofessional communication, the effectiveness of the current paging system, the types of patient care situations for which paging is important, and the challenges faced when using paging to communicate with surgeons. Residents’ interviews will explore their perspectives on interprofessional communication, the effectiveness of the current paging system, types of patient care situations for which paging is appropriate, and current challenges faced with receiving and responding to pages from the general surgery ward. Questions will elicit each professional’s positive and negative experiences with paging and ideas for optimizing the use of this system and interprofessional communication more broadly. Interviews will be conducted by an experienced qualitative researcher for approximately 30 minutes (Multimedia Appendix 2: Interview Guide), audiorecorded, and transcribed.

Nurses and residents will be individually shadowed by a trained observer. Shadowing will take place at varying times during the day and overnight, to capture the range of patient care activities and clinical scenarios in which nurses page surgeons and residents receive pages. During shadowing sessions, the observer will take handwritten notes to document communication as it pertains to nurses and surgeons’ collaborative patient care. These notes will be transcribed into documents comprising reconstructed reflective field notes [22]. All field notes will be anonymized and no identifiable patient information will be recorded. Shadowing sessions will last 2-3 hours each.

Analysis of interview and shadowing data will be inductive and iterative. We will use emerging themes to generate questions for participants as the project progresses, by transcribing, reading, coding, and comparing data in cyclical fashion, identifying recurring themes and ideas as well as disconfirming cases. A constant validity check will be used to guide data interpretation. This entails identifying and exploring participants’ opposing perspectives and ideas, member-checking (ie, asking participants to confirm data), searching for negative evidence, searching for alternative explanations, and theorizing negative cases.

Data from nurse and surgeon interviews and shadowing will be triangulated to produce a holistic description and analytic interpretation of nurse-surgeon paging and response experiences. A comparative analysis of perspectives and experiences of nurses and house staff between the two sites will be conducted.

### Survey of Attitudes and Satisfaction With Interprofessional Communications

We will conduct a survey to explore attitudes about the effectiveness and quality of communication, and assess the overall satisfaction with paging as a means of communication and the reasons for satisfaction or dissatisfaction. The survey will allow for confirmation or of the conclusions drawn from, by assessing perceptions of a larger number of professionals. It will also investigate further potential new hypotheses or information that will emerge from the quantitative analysis.

The survey will be distributed to all general surgery nurses and residents working at SHSC, including those who will have participated in the interviews and shadowing assessments. Potential participants will be identified through the SHSC Department of Nursing and Division of General Surgery. We estimate that 50 nurses and 15 residents will be surveyed.

A group of experts will identify important domains and specific issues within those domains to be addressed in the survey, highlighting those most pertinent interprofessional communications. Items will initially be generated without restriction through inductive analysis by the experts, informed by the results from the previous quantitative, content, interviews, and shadowing analyses. The generated items will then be grouped into domains. Finally, the list of items will be reduced to eliminate redundancy and keep only the most relevant items. To this end, the experts will be asked to rate the relevancy of each time on a 1 to 5 Likert scale [23]. Closed questions using 4 to 5 level Likert scales will be constructed to assess the perceptions of respondents. In addition, we will solicit feedback on fifteen actual verbatim pages selected by the group of experts, with an effort to well represent the range of urgency and subject matter. Scales will be used for respondents to rate each page for clarity, urgency, overall amount of time within which a call-back should occur, and overall amount of time within which medical intervention should occur. Respondents will also have an opportunity to provide brief comments on the pages via an open-ended question.

To assess the clarity and interpretation of the questionnaire, it will be pretested among four residents and four nurses. They will be asked to provide feedback about the flow, clarity, and ease of administration of the questionnaire. The expert group will evaluate face validity, clarity, and comprehensiveness trough a clinical sensibility analysis [23]. The questionnaire will be revised accordingly.

The survey will be self-administered trough a Web-based platform (Fluidsurveys, Chide.it Inc, Ottawa, Canada). Each potential respondent will receive an individual invitation to complete the survey, through their institutional email address. Electronic reminders will be sent 3 and 6 weeks after the initial invite. Gift certificates will be drawn among all respondents, as an incentive to improve response rate.

We will first perform a descriptive analysis of completed questionnaires. Responses will be reported using proportions (n/N, %), and median with inter-quartile range for scale responses. Responses from nurses and house staff will be compared using the chi-square or ANOVA test. Comparison between responses obtained at the two sites will be compared using stratified chi-square and ANOVA. Regression analyses will be conducted, including the site as a covariable. All *P* values less than .05 will be considered statistically significant.

## Discussion

Few studies have investigated the profile of pages to surgical services. Describing the profile of pages is the first step towards identifying areas of inefficiency within the hospital, thereby allowing for the improvement of both the quality of patient care as well as the educational experience of all general surgery residents.

Qualitative methods have proven to be well-suited to uncovering the root causes of what are oftentimes deeply embedded contextual barriers to quality improvement and safety implementation [24]. The comparative design of the qualitative and survey analyses will allow for comparison and contrast of the contexts in which certain interprofessional communication practices, such as paging, are favorable or detrimental to quality patient care. Our mixed methods approach will provide important insights to the type and quality of interprofessional communication and collaboration within general surgery. This issue is important from a broad systems perspective as the results from this study will ultimately lead to the discovery of key contextual barriers to nurse-surgeon communication. It will identify existing interprofessional knowledge and practice gaps, and it will inform the design of improvement efforts to address them.

In order to highlight contextual barriers and facilitators to optimal interprofessional communication and to generate input and buy-in for addressing these barriers, our findings will be disseminated at the organizational level and through nursing teams and surgery rounds. Additionally, results from this study will inform the development of a tailored consensus guideline and communication protocol for general surgery, aimed at improving interprofessional communications. Institutional executives, program leaders, and managers who are part of the research team will support implementation of this protocol, which will ensure successful translation of our findings into practice. Results of this strategy will be evaluated 2 years after completion of its implementation using the same mixed-methods approach to quantitative and qualitative appraisal of interprofessional communication, and comparing it with the current results in a before-and-after design.

## Multimedia Appendix 1

Assessment of Pages Communication Priority based on the Institutional Escalation Response Time Clinical Practice Guideline (Sunnybrook Health Sciences Centre).

## Multimedia Appendix 2

Sample Interview Guides (not exhaustive).

## Multimedia Appendix 3

Funding agency letter confirming funding.

## Abbreviations

ANP: alphanumeric page
IPC: interprofessional communication
SHSC: Sunnybrook Health Sciences Centre
