# Supplementary material for: Optimizing Inter-Professional Communications in Surgery: Protocol for a Mixed-Methods Exploratory Study
Source: JMIR Res Protoc. 2015 Mar 5;4(1):e8. doi: 10.2196/resprot.3623 (PMC4376151; doi:10.2196/resprot.3623)
Supplement: Supplementary file 1 [file resprot_v4i1e8_app1.pdf]

Appendix 1: Assessment of Pages Communication Priority based on the Institutional Escalation Response Time Clinical Practice Guideline (Sunnybrook Health Sciences Centre)

Communication Priority	Approximate call-back time range	Definition	Examples
Low	> 25 minutes	Medical intervention can occur after **more than 120 minutes** without harm for the patient.	
Medium	15-25 minutes	1. Less important and time sensitive; AND<br>2. Medical intervention should likely occur within the **next 120 minutes** or serious harm may result to patient.	New requirement for Oxygen to maintain Oxygen saturation or resolve dyspnea<br>New confusion or agitation<br>New bleeding (non-brisk) with normal vital signs e.g. from a wound<br>Uncontrolled pain despite using existing orders for pain management
High	5-15 minutes	1. Urgent and important; AND<br>2. Medical intervention should likely occur within the **next 30 minutes** or serious harm may result to patient.	Altered vital signs<br>New onset chest pain or difficulty breathing unresolved with supplemental oxygen<br>New alteration of consciousness<br>Brisk bleeding or bleeding with altered vital signs e.g. from a wound
Immediate Emergency Code		1. Urgent and important; AND<br>2. **Immediate medical intervention** should likely occur or serious harm or death may occur.	Rapid Response Team<br>Code Blue (adult cardiac arrest)<br>Code Blue Child<br>Code Pink (pediatric cardiac arrest)<br>Code Omega (massive

transfusion protocol)
