# Supplementary material for: Optimizing Inter-Professional Communications in Surgery: Protocol for a Mixed-Methods Exploratory Study
Source: JMIR Res Protoc. 2015 Mar 5;4(1):e8. doi: 10.2196/resprot.3623 (PMC4376151; doi:10.2196/resprot.3623)
Supplement: Supplementary file 2 [file resprot_v4i1e8_app2.pdf]

Appendix 2: Sample Interview Guides (not exhaustive)

Guide I: Nurses

1. Can you describe a typical scenario when you would page a surgeon?
2. What factors do you take into consideration when you are thinking about paging a surgeon?
3. On average, how many times per day do you send a page to a surgeon?
4. What do you think influences the rate at which surgeons respond to a page?
5. Are there other ways that you communicate with surgeons during the day about patient care?
6. What do you think is the most important aspect of communication between nurses and surgeons when caring for patients?
7. What, if anything, do you feel can be improved about the paging communications that happen between nurses and surgeons?

Guide II: Surgeons

1. Can you describe a typical scenario for which you would receive a page from a surgery nurse?
2. What factors do you take into consideration when you are thinking about responding to a page?
3. On average, how many times per day do you receive a page from a surgery nurse?
4. What do you think influences the rate at which nurses send pages to surgeons?
5. Are there other ways that you communicate with nurses during the day about patient care?
6. What do you think is the most important aspect of communication between nurses and surgeons?
7. What, if anything, do you feel can be improved about communication between nurses and surgeons?
