# Supplementary material for: Optimizing Inter-Professional Communications in Surgery: Protocol for a Mixed-Methods Exploratory Study
Source: JMIR Res Protoc. 2015 Mar 5;4(1):e8. doi: 10.2196/resprot.3623 (PMC4376151; doi:10.2196/resprot.3623)
Supplement: Supplementary file 3 [file resprot_v4i1e8_app3.pdf]

Dr. Natalie Coburn
Sunnybrook Health Sciences Centre

March 7, 2014

**NEW: 2013-2014 Sunnybrook Innovation Funding Contract**

Dear Dr. Coburn,

We are pleased to inform you that your proposal to the Sunnybrook AFP Innovation Fund Review Committee, titled *Optimizing Inter-professional Communication between Surgical Nurses and House Staff: An Exploratory Study,* has been approved for funding by the Innovation Fund Provincial Oversight Committee in the amount of $ 99.986.

We expect funds will flow with the March 31 AFP payments, and if this timeline holds, Sunnybrook AFP will transfer your Innovation Funds to Sunnybrook Research Institute (SRI) in April. **It is imperative that you sign the attached form within 15 days** in order to open your account with SRI in time to accept the funds.

You are also required to sign and return this letter to confirm that you have reviewed and accept the terms and the conditions of funding as follows:

1. Your project funding is for a two year project with an end date of May 30, 2016.
2. You will submit an Interim Project Report including an Interim Budget Report to the Sunnybrook AFP within 12 months of receiving the funds. Due May 30, 2015.
3. You will submit a Final Project Report and Final Budget Report within two years of the end date of your project. Due May 30, 2018. At this time your project will be considered complete.
4. Projects will be managed by project leads.
5. Funds must be administered by Sunnybrook Research Institute.
6. Expenditures must meet Innovation Fund Provincial Oversight Committee (IFPOC) Framework requirements and be consistent with submitted budgets.
7. Project leads who do not submit Interim and Final reports, or acceptable reasons why they are unable to within the timelines specified will have their AFP Innovation Funds frozen.
8. Projects that do not provide reports as required, will be required to return the funds in full to Sunnybrook AFP, who will return the funds to the Ministry.
9. Unspent funds for completed projects will be returned to Sunnybrook AFP who will return the funds to the Ministry.
10. You understand that the Sunnybrook AFP Board is responsible to the Ministry of Health and Long Term Care for ensuring that AFP Innovation funds are appropriately managed and used

for their intended purpose. Failure to fulfill your reporting commitments could jeopardize your AFP Funding and that of your practice plan and department should it be necessary for the Board to recover the funding.

11. Project publications will acknowledge AHSC AFP Innovation Funding from the MOHLTC. The following acknowledgement is recommended: "____was supported by the Innovation Fund of the Alternative Funding Plan from the Academic Health Sciences Centres of Ontario."
12. Funds will be spent within the two year time frame of the project and by the end date May 30, 2016.

Please contact Lynne Bridgman or Asa Jairam in the AFP office if you have questions or need assistance completing the forms. Once you have completed, signed and returned this letter and the attached account opening form, we will be in touch with more details regarding the release of the funding and reporting requirements.

Congratulations on your successful submission.

Regards,

Dr. Richard M. Holtby
*Chair of the Board*
*Sunnybrook Medical Services Alternative Funding Plan Association*

**This is to confirm that I have reviewed and will comply with terms and conditions of AFP Innovation Funding including those outlined above.**

______________________ ______________

**Dr. Natalie Coburn,** *Project Lead* Date

**I have read and understand the implications of non-compliance as outlined in point #7 &10 above.**

______________________ ______________

**Dr. Avery Nathens,** *Surgeon-in-Chief* Date

______________________ ______________

**Dr. Peter Chu** Date
*Financial Unit Manager, Surgery*
